# Genome plasticity favours double chromosomal Tn*4401b*-*bla*_KPC-2_ transposon insertion in the *Pseudomonas aeruginosa* ST235 clone

**DOI:** 10.1186/s12866-019-1418-6

**Published:** 2019-02-20

**Authors:** Deisy Abril, Ricaurte Alejandro Marquez-Ortiz, Betsy Castro-Cardozo, José Ignacio Moncayo-Ortiz, Narda María Olarte Escobar, Zayda Lorena Corredor Rozo, Niradiz Reyes, Catalina Tovar, Héctor Fabio Sánchez, Jaime Castellanos, Yina Marcela Guaca-González, Carmen Elisa Llanos-Uribe, Natasha Vanegas Gómez, Javier Escobar-Pérez

**Affiliations:** 1 Bacterial Molecular Genetics Laboratory, Universidad El Bosque, Carrera 9 N°131A-02, Bogotá D.C, Colombia; 2 Grupo de Investigación en Enfermedades Infecciosas- GRIENI, Facultad de Ciencias de la Salud, Universidad Tecnológica de Pereira, Pereira, Colombia; 3 Hospital El Tunal E.S.E, Bogotá D.C, Colombia; 4 Grupo de Genética y Biología Molecular, Universidad de Cartagena, Cartagena, Colombia; 5 Grupo de Investigación en Enfermedades Tropicales y Resistencia Bacteriana, Universidad del Sinú, Montería, Colombia; 6 Hospital Universitario Departamental de Nariño, Pasto, Colombia; 7 Grupo de Patogénesis Infecciosa, Universidad Nacional de Colombia, Bogotá D.C, Colombia; 8 ESE-Hospital Universitario San Jorge, Pereira, Colombia; 9 The i3 institute, Faculty of Science University of Technology, Sydney, Australia

**Keywords:** *bla*_KPC-2_, *Pseudomonas aeruginosa*, Carbapenems, Resistance, Colombia, ST235

## Abstract

**Background:**

*Pseudomonas aeruginosa* Sequence Type 235 is a clone that possesses an extraordinary ability to acquire mobile genetic elements and has been associated with the spread of resistance genes, including genes that encode for carbapenemases. Here, we aim to characterize the genetic platforms involved in resistance dissemination in *bla*_KPC-2_*-*positive *P. aeruginosa* ST235 in Colombia.

**Results:**

In a prospective surveillance study of infections in adult patients attended in five ICUs in five distant cities in Colombia, 58 isolates of *P. aeruginosa* were recovered, of which, 27 (46.6%) were resistant to carbapenems. The molecular analysis showed that 6 (22.2%) and 4 (14.8%) isolates harboured the *bla*_VIM_ and *bla*_KPC-2_ genes, respectively. The four *bla*_KPC-2_-positive isolates showed a similar PFGE pulsotype and belonged to ST235. Complete genome sequencing of a representative ST235 isolate shows a unique chromosomal contig of 7097.241 bp with eight different resistance genes identified and five transposons: a Tn*6162-like* with *ant(2″)-Ia*, two Tn*402-like* with *ant(3″)-Ia* and *bla*_OXA-2_ and two Tn*4401b* with *bla*_KPC-2_. All transposons were inserted into the genomic islands. Interestingly, the two Tn*4401b* copies harbouring *bla*_KPC-2_ were adjacently inserted into a new genomic island (PAGI-17) with traces of a replicative transposition process. This double insertion was probably driven by several structural changes within the chromosomal region containing PAGI-17 in the ST235 background.

**Conclusion:**

This is the first report of a double Tn*4401b* chromosomal insertion in *P. aeruginosa*, just within a new genomic island (PAGI-17). This finding indicates once again the great genomic plasticity of this microorganism*.*

**Electronic supplementary material:**

The online version of this article (10.1186/s12866-019-1418-6) contains supplementary material, which is available to authorized users.

## Background

*Pseudomonas aeruginosa* is an opportunistic pathogen that causes numerous infections in hospitalized patients, especially in the intensive care unit (ICU), some of which are difficult to treat because of their multidrug-resistant (MDR) phenotype. In *P. aeruginosa* carbapenem resistance has been associated mainly with two different mechanisms: first by preventing the access of the antibiotic to its active site by means of chromosomal mutations that upregulate of multidrug efflux systems or alterations in the outer membrane permeability and second by the acquisition and production of carbapenemases [1]. In the last case, the most frequent carbapenemases found in *P. aeruginosa* are GES, IMP, VIM, SPM and more recently KPC and NDM [1–6].

The KPC enzyme is the most frequent class A carbapenemase and has been reported in countries from five continents. KPC has been found mainly in the epidemic *Klebsiella pneumoniae* ST258 clone and in some *Enterobacteriaceae* [7]; however, it has also been identified in *P. aeruginosa*, first in Colombia [2], then in Trinidad and Tobago [8], the United States of America [3], Puerto Rico [9], China [10] and Brazil [11]. The *bla*_KPC_ gene (that encodes the KPC enzyme) is mobilized on Tn*4401*, an active transposon belonging to the Tn*3* family, with a size of 10 kb, that is delimited by two 39-bp inverted repeat sequences, and its replicative transposition involves a 5-bp target site duplication, without target site specificity [12]. Currently, eight different isoforms of Tn*4401* have been reported (a-h), which differ by size-variable deletions upstream at the *bla*_KPC_ gene, truncated *tnpA* or lacking *tnpR* [13, 14]. In *P. aeruginosa,* the *bla*_KPC_ gene is rare (with variant 2 being the most frequent) and is mainly mobilized on both complete or truncated Tn*4401b* transposons, which have been reported in two different plasmid backbones (8 kb and 31.5 kb) belonging to the IncU and IncP-6 incompatibility groups, respectively [15, 16]. This *bla*_KPC-2_ variant has been identified in *P. aeruginosa* isolates belonging to sequence type (ST) 308, 1006, 1060 and 235 [15–17].

*Pseudomonas aeruginosa* ST235 is an infectious and virulent clone that possesses an extraordinary ability to acquire mobile genetic elements (MGE) and has been associated with the spread of multiple resistance genes, including genes that encode for carbapenemases [18–20]. Currently, this clone has been identified across the world, showing great disseminative success [18, 19, 21, 22]. The genomic plasticity of *P. aeruginosa* can be seen in ST235 strains, where the content of the resistance genes varies greatly due to the capture, permanence and rearrangement of resistance regions [18, 23, 24]. In Colombia, the ST235 clone has become prevalent since 2005 and has suffered an evolution in clinically important resistance genes by lateral gene transfer (LGT), e.g. some strains have acquired an atypical active class 1 integron with an unusual 3′ consensus sequence (*tni* module), which can provide a putative self-transposition ability [25], the acquisition of IS*26* and the *bla*_KPC-2_ gene, which are most frequent in *Enterobacteriaceae* but rare in *P. aeruginosa* [2, 25]. In this study, after active clinical surveillance in the ICU at five distant cities, we found a broad population distribution of *P. aeruginosa* in Colombia; nonetheless, the ST235 clone was the only one harbouring *bla*_KPC-2_, and interestingly, those ST235 isolates harboured two copies of this gene that were located in two Tn*4401b* transposons in a new genomic island (GI) at the chromosome. As far as we know, this is the first report of a double chromosomal *bla*_KPC-2_ insertion in *P. aeruginosa*.

## Methods

### Strain collection, susceptibility profile and PCR detection of resistance genes

This prospective study was carried out based on the active surveillance of *P. aeruginosa* infections in adult patients who were attended in five ICUs in five distant cities in Colombia between October 2014 and March 2016. A total of 58 *P. aeruginosa* isolates were recovered. The susceptibility profiles of the isolates to meropenem, imipenem, ertapenem and doripenem, ceftazidime, gentamicin, amikacin, ciprofloxacin, trimethoprim/sulfamethoxazole, and Piperacillin/Tazobactam were determined by the automated systems VITEK® 2 or Microscan®. Additionally, the minimum inhibitory concentration (MIC) was established by the broth dilution method. The susceptibility profiles were established using the breakpoints defined by the Clinical and Laboratory Standards Institute according to the 2017 update [26]. The ATCC 9721 *P. aeruginosa* strain was used as control. In addition, the following resistance genes were assessed in all the isolates: *bla*_TEM_*, bla*_SHV_*, bla*_CTX-M_*, bla*_FOX_, *bla*_ACT_, *bla*_MIR_ *bla*_ACC_, *bla*_DHA_, *bla*_CMY_, *bla*_MOX_, *bla*_IMP_*, bla*_OXA-48_*, bla*_VIM_*, bla*_GES_*, bla*_KPC_*, bla*_NDM_*, bla*_OXA-23_, *bla*_OXA-24_, *bla*_OXA-43_, *bla*_OXA-51*,*_ *bla*_OXA-58_, *aac(6′)-lb, aac(6′)-lb-cr, qnrA, B y S, mcbg, sul1, sul2* and *sul3.* The *bla*_KPC-2_ variant was determined by digestion with *Rsa*I enzyme and confirmed by sequencing. Finally, the *intl1, intl2* and *intl3* genes were assessed.

### Phylogenetic relationship among *Pseudomonas aeruginosa* isolates

The genetic relatedness between the isolates was determined by genome macro-restriction using the *Spe*I enzyme (Promega) and PFGE separation according to the protocol reported by Herschleb et al [27]. The dendrogram was obtained using the GelCompar II program (Applied Maths NV) with a tolerance position of 1.5% and a Dice coefficient of 1.0%. Two pulsotypes were considered different when they had < 80% similarity. The sequence type (ST) to some representative isolates of the most frequent pulsotypes was determined using the protocol reported by Curran et al [28].

### Whole genome sequencing of a ST235 *bla*_KPC-2_-positive *Pseudomonas aeruginosa* isolate

The complete genome sequence of the ST235 *bla*_KPC-2_*-*positive 24pae112 isolate was obtained using the PacBio RS II platform (Pacific Biosciences, USA) and assembled through the previously reported procedure [29]. In brief, the total DNA was extracted, and a BluePippin (Sage Science) 20-kb size-selected library was prepared and then sequenced using one SMRT cell on the PacBio RS II platform. The sequencing process of the 24Pae112 isolate yielded 136,958 reads and a total of 1,221,367,395 bases (coverage of 172X). Sequencing reads were de novo assembled using the HGAP 3 protocol with default parameters. The assembly was visually inspected and manually verified using BWA-MEM (Burrows-Wheeler Aligner with maximal exact matches) [30] and Tablet v1.15.09.01 [31]. Misassembled terminal repeat overlap sequences were identified with Gepard (Genome Pair Rapid Dotter) [32] and trimmed manually. The whole genome was annotated using Prokka v1.11 [33], and the interesting regions were manually confirmed using BLASTn and BLASTp and edited in Artemis [34].

### Phylogenetic analysis and comparative genomics of ST235 isolates

To perform the phylogenetic analysis and comparative genomics, the 43 publicly available *P. aeruginosa* ST235 genomes at the time were included (41 drafts and 2 complete genomes available at the NCBI genomes repository in January 2017), plus the genomes of the 24pae112 and 9Ps50 isolates (Additional file 1: Table S1). This 9Ps50 isolate was previously identified in 2006 by our group, causing an infection in institution C (included in this study), and did not harbour *bla*_KPC-2_ [25]. To build the phylogenetic tree based on the core-genome SNPs, partially assembled genomes were annotated using Prokka v1.11 [33], and an alignment of the concatenated core genes (genes present in all genomes with ≥90% of nucleotide identity) was created with Roary [35] using PRANK [36]. Poorly aligned positions and divergent regions were eliminated using Gblocks [37]. The phylogenetic tree was created using RAxML version 8.2.9 [38] running 1000 bootstrap replicates under the generalized time reversible model (GTRCAT). Finally, the consensus tree was plotted using Dendroscope [39]. Branch lengths are expressed in changes/nucleotide position (scale bar). In addition, an accessory-genome-based phylogenetic tree was built (presence/absence of genes) to assess the genetic diversity of the foreign DNA into the ST235 clone using the programs mentioned above.

A genomic comparative analysis was performed to determine the genetic variability in all *P. aeruginosa* ST235 strains using BLASTn [40] and visualized with BRIG. Genomic and pathogenicity islands (GI and PI, respectively), prophages, insertion sequence (IS) elements and transposons were identified using IslandViewer [41], PHASTER [42] and ISfinder [43]. The genetic platforms identified were visualized and illustrated by Artemis [34], ACT [44] and EasyFig [45].

### Resistome identification

To determine the resistance gene content variability in the *P. aeruginosa* ST235 clone, the antibiotic resistance genes were identified in the genome of all the sequenced ST235 isolates using ARIBA (https://github.com/sanger-pathogens/ariba/wiki), with the ResFinder [46], CARD (The Comprehensive Antibiotic Resistance Database) [47] and ARG-ANNOT (Antibiotic Resistance Gene-ANNOTation) [48] databases. The results were visualized through Phandango software.

## Results

**Fig. 1:**
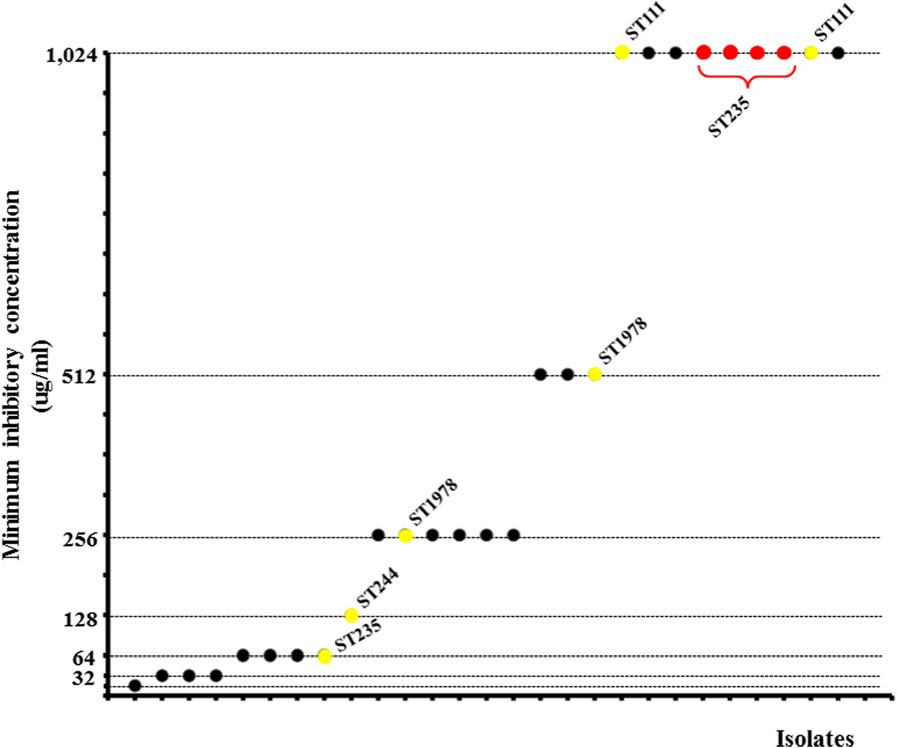
Behaviour of the minimum inhibitory concentration (MIC) to meropenem of the 27 carbapenem-resistant isolates. Yellow and red points indicate the VIM-producing and KPC-producing isolates, respectively. ST: Sequence type

In the surveillance study, we identified 58 *P. aeruginosa* that caused ICU infections among adult patients. With respect to the resistance behaviour, among all 58 isolates, 27 (46.6%) were resistant to at least one carbapenem antibiotic (imipenem, meropenem, ertapenem or doripenem). Of these 27 carbapenem-resistant *P. aeruginosa*, six (22.2%) and four (14.8%) isolates contained the *bla*_VIM_ and *bla*_KPC-2_ genes, respectively. Multilocus sequence typing shows that the six *bla*_VIM_-positive isolates belonged to four different ST’s: ST111 (two isolates) previously reported in other Colombian cities [17]; ST244 (one isolate) reported in Europe [49, 50], Asia [21] and Africa [51]; ST1978 (two isolates), which was not reported previously; and ST235 (one isolate). Interestingly, all *bla*_KPC-2_-positive isolates only belonged to ST235. Analysis of the meropenem MIC showed a heterogeneous behaviour among the carbapenem-resistant isolates. Nine isolates reached an MIC value of 1024 μg/ml; among them were all the *bla*_KPC-2_-positive ST235 isolates and two *bla*_VIM_-positive ST111 isolates. Curiously, three isolates had an MIC of 1024 μg/ml but did not contain any of the assessed carbapenemases genes (Fig. 1).

The PFGE analysis revealed 34 pulsotypes in the 58 *P. aeruginosa* isolates, showing a polyclonal behaviour. The six VIM-producing isolates were found in four unrelated PFGE pulsotypes, each of them with a different ST, suggesting that diverse clones have acquired this carbapenemase (Additional file 2: Figure S1). In contrast, in the four KPC-2-producing isolates (24Pae65, 24Pae112, 24Pae153 and 24Pae250), two very similar PFGE pulsotypes (a unique band of difference, > 95% of similarity) were found (Additional file 2: Figure S1).

### Genomic comparative analysis of the ST235 isolates

**Fig. 2:**
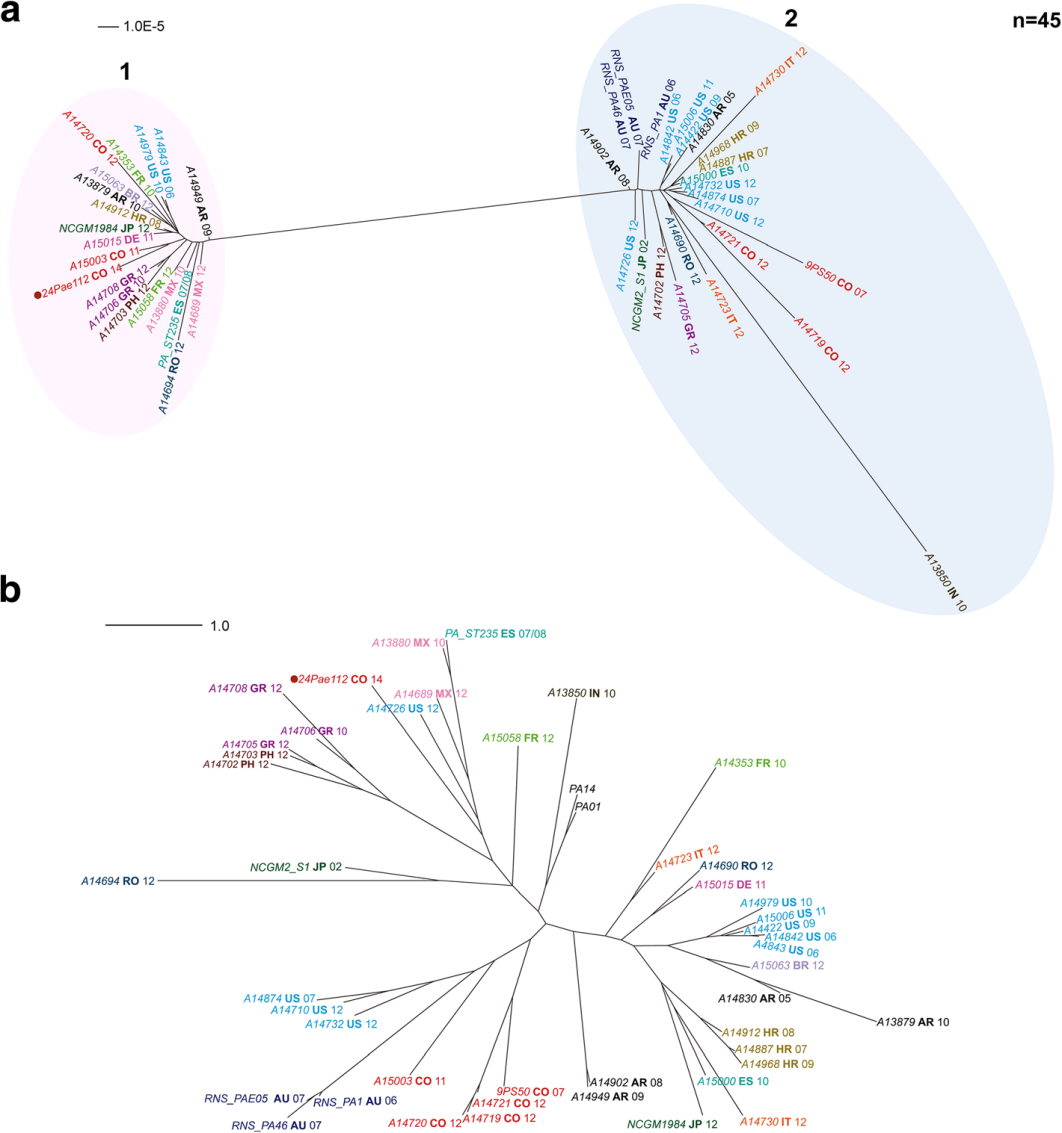
Maximum likehood phylogenetic tree based on core (**a**) and accessory (**b**) genome of the 45 ST235 isolates. The isolates are named as follow: strain name, acronym of the origin country in bold (see Additional file 1: Table S1) and date of identification. The strains that start as AZPAE1 were simplified in the figure as A1. The branches into the tree were established when the sum of all internal nodes were ≤ 2, every branch is show in different colours. The bar scale (1.0 and 0.001) indicate the SNP number

In order to go deeper into the genetic platforms mobilizing the *bla*_KPC-2_ and other resistance genes, the complete genome sequence of the 24Pae112 isolate was established. After PacBio sequencing and assembly process, the 24Pae112 genome had a size of 7097,245 bp, in a unique contig (suggesting that this isolate does not contains plasmids), with a 66.0% G + C content, comprising 6634 predicted genes (coding density of 88.2%), which include 328 (4.9%) membrane transport proteins, 224 (3.4%) cell wall synthesis proteins, 202 (3.0%) virulence factors (pathogenicity and defence), 97 (1.5%) proteins of mobile genetic elements (prophages, insertion sequences and transposons), 63 (0.9%) tRNAs and 12 (0.2%) rRNAs. The antimicrobial resistome to the 24Pae112 chromosome evaluated using ARIBA included resistance genes to aminoglycosides (*ant(2″)-Ia, ant(3″)-Ia* and *aph(3′)-IIb*), beta-lactams (*bla*_*OXA-2*_ and *bla*_*KPC-2*_), sulphonamides (*sul1*), chloramphenicol (*catB7*) and fosfomycin (*fosA*). In addition, 8 prophages, 7 genomic islands (GI) and the different transposons within them were identified in the genome (Table 1). Recently, 40 and three ST235 genomes (complete or draft) were reported at the public NCBI genome database from the different countries, and we performed a phylogenetic analysis based on the core genome SNPs to determine the relationship between them and the Colombian ST235 isolates (Fig. 2). This analysis reveals that ST235 can be divided in two different genetic clades (Clade 1 and 2), showing the circulation of two genetic “sublineages” within the ST235 clone. No phylogenetic correlation with the origin country or identification date could be demonstrated because several isolates do not cluster according to their geographic origin or the date when they were identified, e.g. of the nine isolates identified in the USA, seven and two were grouped in clade 2 and 1, respectively; the six Colombian isolates were grouped in each of the clades. Meanwhile, the 24Pae112 and 9Ps50 isolates were grouped in different clades. These results suggest that two genetic “sublineages” are circulating within the ST235 clone. Interestingly, the accessory-genome-based tree of the ST235 clone contained more branches than the core-genome-based tree, showing a relatively wide genetic variability in the accessory genome of the ST235 isolates. The gene content on the accessory genome of the 24Pae112 isolate was closely related to the AZPAE14726 isolate that was identified in the USA in 2012, and very distant to the other Colombian ST235 isolates suggesting that the 24Pae112 isolate acquired a very different accessory DNA, perhaps by differential LGT events.

**Table 1:** Genomic islands and prophages present in the *Pseudomonas aeruginosa* 24Pae112 genome

Genomic island (GI) or prophage (PP)	Start	End	Size (bp)	GenBank accession number^a^	% of 24Pae112 coverage	% of Identity
H70 1st copy (PP)	262,871	300,420	37,549	NC_027384	80	91
H70 2nd copy (PP)	5,678,768	5,716,362	37,594	NC_027384	82	91
PM105 1st copy (PP)	2,541,628	2,580,611	38,983	NC_028667	79	98
PM105 2nd copy (PP)	2,940,222	2,979,923	39,701	NC_028667	82	97
D3 (PP)	3,879,471	3,936,450	56,979	NC_002484	44	95
F10 (PP)^b^	5,053,812	5,092,700	38,888	NC_007805	13	94
phiCTX (PP)	705,252	724,278	19,026	NC_003278	2	78
Pf1 (PP)	5,347,267	5,358,785	11,518	NC_001331	80	98
PAGI-1 (GI)	3,366,957	3,406,228	39,271	AF241171	76	99
PAGI-2 (GI)	2,310,022	2,363,289	53,267	AF440523	33	99
PAGI-4 (GI)	4,795,506	4,895,009	99,593	AY258138	61	99
PAGI-5 (GI)^b^	5,752,816	5,853,757	100,941	EF611301	70	99
PAGI-6 (GI)	6,593,897	6,630,920	37,023	EF611302	66	97
GI1 (GI)^b^	2,403,928	2,472,004	68,076	EU595750	91	99
PAGI-17 (GI)^b^	2,724,254	2,841,225	116,971	NC_018080	90	99

^a^GenBank accession number of sequence used as reference

^b^Harbouring transposons with resistance genes

### *Pseudomonas aeruginosa* ST235 resistome

**Fig. 3:**
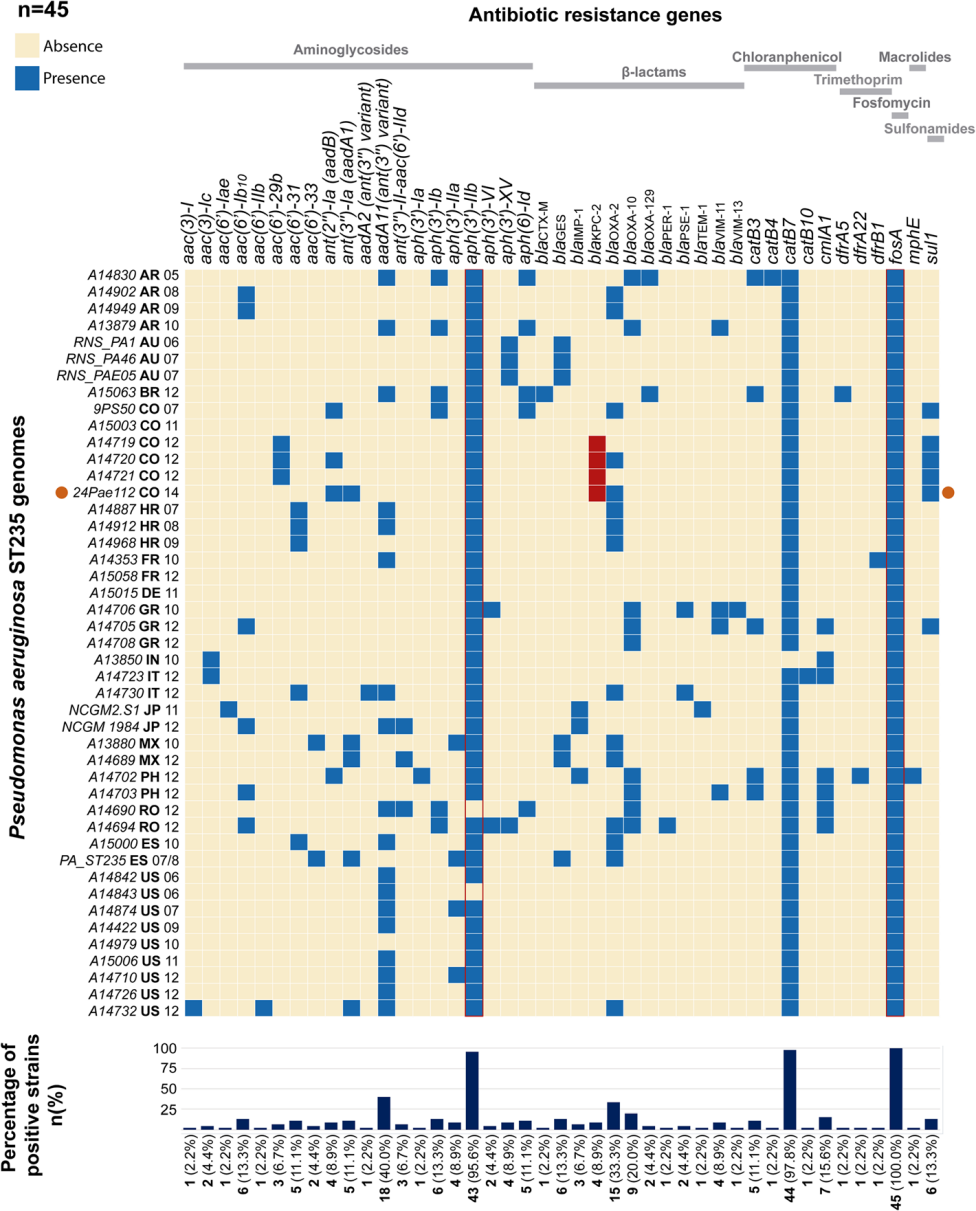
Resistome of *Pseudomonas aeruginosa* ST235 clone. Resistance genes identified in the strains belonging to ST235. The blue and red squares represent the presence of the resistance genes and the isolates harbouring the *bla*_KPC_ gene, respectively. The isolates are named as follow: strain name, acronym of the origin country in bold (see Additional file 1: Table S1) and date of identification. The orange circle indicates the position of the 24Pae112 isolate

The *P. aeruginosa* ST235 clone is recognized for its ability to acquire and spread resistance genes worldwide. We determined the resistance genes repertoire in the 45 ST235 genomes included in the study. Multiple resistance genes to different families of antibiotics were found (Fig. 3). Of note, five different genes related to carbapenem resistance were found distributed in 8 countries: *bla*_GES_, *bla*_KPC-2_, *bla*_VIM-11_, *bla*_IMP-1_, and *bla*_VIM-13_ in 10 (22.2%), 4 (8.9%), 4 (8.9%), 3 (6.7%) and 1 (2.2%) isolates, respectively. Nonetheless, the gene *bla*_KPC-2_ (the unique variant detected in this clone) was exclusively found in the Colombian ST235 isolates, showing that the acquisition of this gene by the ST235 clone may be a particular phenomenon in our country. In addition, 22 gene variants related to aminoglycoside resistance were identified. The *qnr*, *mcbg* and *aac(6′)-Ib-cr* genes related to plasmid-mediated quinolone resistance were not found.

### Diverse genetic platforms transport resistance genes in 24Pae112 isolate

**Fig. 4:**
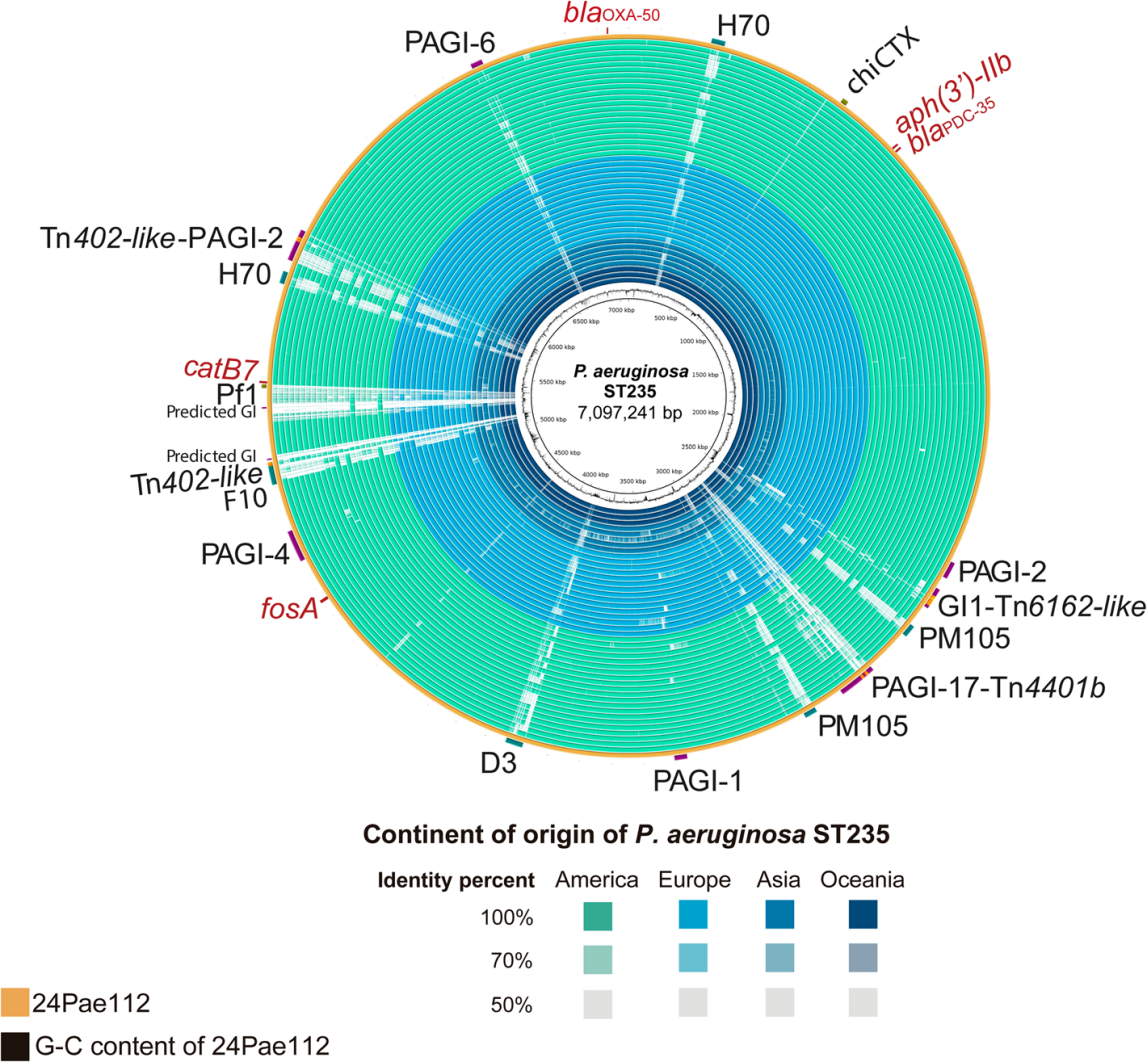
Genomic comparison of *Pseudomonas aeruginosa* ST235 isolates. Matched alignment between 24Pae112 genome and the 40 and four remain ST235 genomes visualized using BRIG, the order of the strains in the rings can be seen in Additional file 1: Table S1. The colours of every ring indicate the continent of the origin and the colour intensity represents the identity percentage. The outer ring shows the location of resistance genes and their genetic platforms of mobilization (when was identified), genomic islands and prophages in 24Pae112

**Fig. 5:**
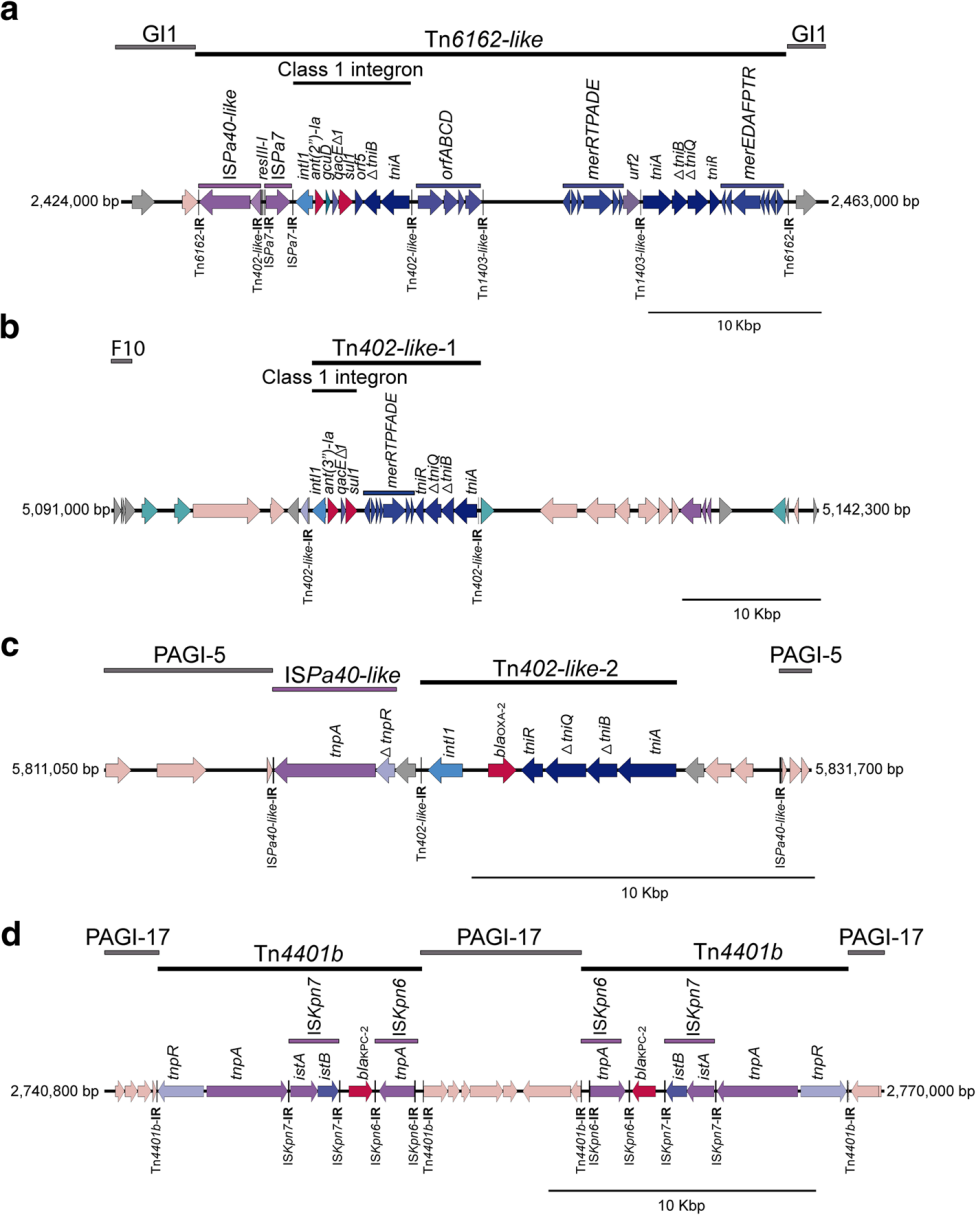
Organization of the different genetic platform harbouring resistance genes within the 24Pae112 isolate. **a** Tn*6162-like* transposon inserted into the genomic island 1. **b** First copy of the Tn*402-like* transposon inserted into the prophage F10. **c** Second copy of the Tn*402-like* transposon within the PAGI-5. **d** Two copies of the Tn*4401* transposon into the new genomic island PAGI-17. IR: Inverted repeats

To gain a better understanding of the complex process of the acquisition of resistance genes by the 24Pae112 isolate, the genetic platforms transporting these genes were identified, and the genome localization was compared with the remaining ST235 genomes and visualized with BRIG (Figs. 4 and 5). Five transposons were identified to harbour resistance genes: the Tn*6162*-like transposon with a size of 34,220 bp, which was located in *P. aeruginosa* genomic island 1 (GI-1). This transposon harbours a classical class 1 integron with the gene cassette array *aadB-gcuD* (Fig. 5a). Sample 24Pae112 also harbours two distant copies of the Tn*402*-like transposon, with the first one in the prophage F10 and the second one in the PAGI-5. Curiously, both transposons contained a class 1 integron structure, but with different variable regions, harbouring only one gene cassette, *aadA1* and *bla*_OXA-2,_ respectively (Fig. 5b and c). In addition, two *bla*_KPC-2_-harbouring Tn*4401b* transposons were also identified (see below). It was not possible to determine the genetic platforms mobilizing the *aph(3′)-IIb, catB7* and *fosA* genes.

### Two *bla*_KPC-2_-harbouring Tn*4401b* are inserted into the 24Pae112 chromosome

**Fig. 6:**
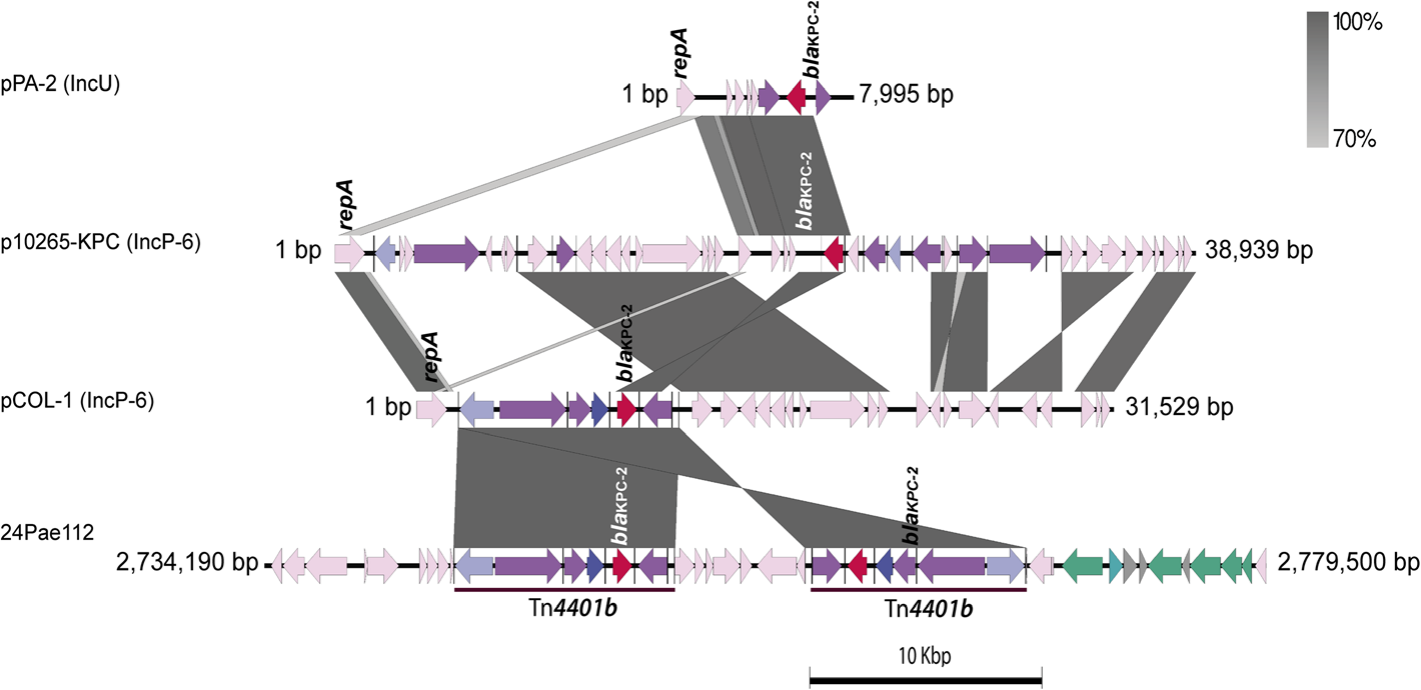
Comparison of the different genetics platform mobilizing *bla*_*KPC-2*_ gene in *Pseudomonas aeruginosa.* The pPA-2 (KC609322.1) and pCOL-1 (KC609323.1) plasmids were identified in Colombia; the p10265-KPC plasmid (KU578314.1) was identified in China

**Fig. 7:**
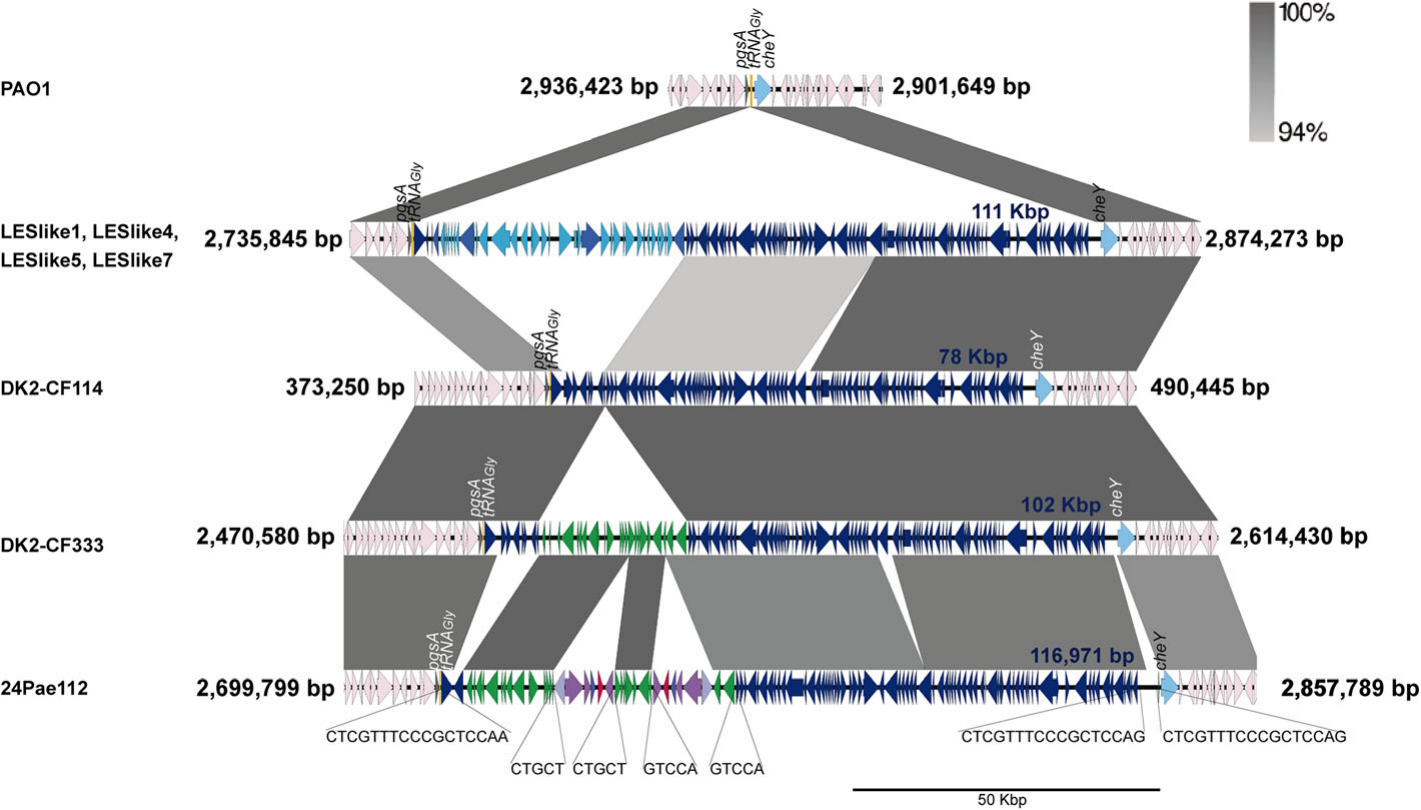
Insertion sites analysis of the two *bla*_KPC-2_-harbouring Tn*4401b* transposons and PAGI-17. The PAGI-17 was inserted at *tRNA*^*Gly*^ gene as it is showed in the PAGI-17-negative PAO1 reference strain (NC_002516.2). The two couples of Tn*4401b* direct repeats and the three direct repeats of the PAGI-17 are showed. The GenBank accession number for DK2, LESlike1, LESlike4, LESlike5 and LESlike7 genomes are NC_018080.1, NZ_CP006984.1, NZ_CP006985.1, NZ_CP006980.1 and NZ_CP006981.1; respectively

Surprisingly, the genome sequence analysis of 24Pae112 reveals the presence of two complete and intact copies of *bla*_KPC-2_-harbouring Tn*4401* (isoform b) transposons inserted into the chromosome within a new genomic island (Figs. 5d and 6). Comparative genomic analysis allowed us to establish that this same genomic island had already been described by Rau et al. in the *P. aeruginosa* DK2 clone and named generically as GI4 [52]; however, the sequence and structure of this island is different with respect to the 16 PAGI currently reported (including PAGI-4, data not shown) [53–57]. Consequently, here, we renamed this genomic island as *Pseudomonas aeruginosa* genomic island 17 (PAGI-17), according to the widely accepted nomenclature system used for genomic islands in this microorganism [53–57]. This double Tn*4401b* chromosomal insertion was experimentally confirmed by PCR using specific primers (data not shown). The two Tn*4401b* copies were identical, with a size of 10,006 bp, in an inverted orientation, for a separate DNA fragment of 5877 bp, which harbours the *parB, yhbS*, *chrA*, *padR* and *arsC* genes. The first Tn*4401b* copy was inserted within the *acr3* gene, which encodes the Acr3 protein related to arsenic resistance [58], with a 5-bp target site duplication (CTGCT). The second Tn*4401b* copy was inserted within the *acrB* gene, which encodes the transporter protein AcrB, related to acriflavine, antibiotics, disinfectants, dyes, and detergent resistance; *AcrB* gene is located upstream of the *acrA* gene which encodes the RND multidrug efflux membrane permease protein AcrA. The second insertion generated a 5-bp distinct target site duplication (GTCCA) (Fig. 7). This confirms the lack of target site specificity of this transposon, as has previously been reported [12].

Although the information about PAGI-17 is very limited, to unravel the genetic mechanisms that possibly allow the Tn*4401b* chromosomal insertion, a more detailed analysis of this genomic island was carried out. Comparing the chromosome sequence of the reference strain *P. aeruginosa* PAO1 (devoid of PAGI-17) with the 24Pae112 genome, we determined that in 24Pae112, the PAGI-17 was inserted at the *tRNA*^*Gly*^ gene, in the positions 2,724,254 to 2,841,225 (to the 24Pae112 genome). The PAGI-17 insertion produced the duplication of a 16-bp sequence (CTCGTTTCCCGCTCCA) located at the 3′-end of the *tRNA*^*Gly*^ gene (Fig. 7). The duplication did not alter the sequence of this gene. The total size of PAGI-17 in 24Pae112 was 116,971 bp, with a G + C content of 64.1% and 113 open reading frames. Comparing PAGI-17 from 24Pae112 with PAGI-17 from *P. aeruginosa* DK2, we identified an additional 16-bp identical sequence (CTCGTTTCCCGCTCCA, similar to that generated by the PAGI-17 insertion in 24Pae112) located downstream of the second duplicated sequence. This new duplication delimits a fragment of a 2264-bp, which is unique in the 24Pae112 PAGI-17 and harbours the *fimA* gene that encodes the type 1 fimbria component protein, an important virulence factor that mediates mannose-sensitive adherence to the eukaryotic cell.

Analysis of the chromosomal environment surrounding the Tn*4401b* insertion sites revealed a 24,134-bp specific DNA fragment, which is rarely found in *P. aeruginosa* since it was only found in PAGI-17 in five unrelated *P. aeruginosa* strains: DK2-CF333(ST386), SCV20265 (ST299), PAER4 (ST260), 8380(ST2619) and BAMCPAO7 (ST313). None of the ST235 isolates contained this region (Fig. 7). While the complete or truncated PAGI-17 is present in several *P. aeruginosa* strains, only six strains (including the 24Pae112) possess this 24,134-bp-size DNA fragment. Interestingly, a genomic comparative analysis carried out by Rau et al. on 45 isolates belonging to the DK2 lineage and recovered from patients suffering from cystic fibrosis over the course of 35 years (from 1973 to 2008) found that this 24,134-bp DNA fragment was acquired early in the DK2 lineage. Specifically, the DK2-CF114 isolate (the first DK2-lineage isolate recovered at 1973) did not contain exactly this fragment, whereas the remaining 44 isolates incorporated it into their chromosomes [52]. This result suggests that this DNA portion was possibly acquired by LGT in *P. aeruginosa* a long time ago in a few genetic lineages, including the ST235 clone. The acquisition of this DNA may favour the incorporation and permanence of the two Tn*4401b*-*bla*_KPC-2_ transposons.

## Discussion

The problem of resistance to carbapenems has become highly relevant for the health care authorities, as this class of antibiotics has frequently been used as the last-line-of-defence agents to treat serious bacterial infections. Additionally, carbapenem resistance is a much bigger problem in non-fermentering bacteria, such as *P. aeruginosa* and *Acinetobacter baumanni,* because these bacteria possess frequent resistance (intrinsic or acquired) to other families of antibiotics, and the lack of available treatment options against some MDR strains is worrying. In our study, we found that almost half (46.6%) of the *P. aeruginosa* isolates were already resistant to carbapenems, which is alarming given that in Colombia the number of MDR *P. aeruginosa* infections in hospitalized patients has risen [59]. High percentages of carbapenem resistance have been reported in other countries, such as Brazil (43.9%), Spain (88.0%) and Iran (96.0%) [60–62]. Additionally, among all the carbapenem resistant isolates, almost 40% are produced by carbapenemase production (VIM and KPC) in different clones (ST111, ST244, ST1978 and ST235). We identified four *bla*_*KPC-2*_-positive and multidrug resistant isolates belonging to ST235 (only susceptible to colistin), but these do not contain the *bla*_*KPC-2*_–harbouring plasmids that were previously reported [15, 16]. For these reasons, we performed complete genome sequencing of the *bla*_*KPC-2*_-harbouring *P. aeruginosa* isolates to establish the different genetic platforms moving the resistance genes. This analysis led us to find a new feature in the dynamic *bla*_*KPC-2*_ gene mobilization through Tn*4401* in this bacterium.

The *bla*_*KPC-2*_ gene in *P. aeruginosa* was first reported in Colombia in 2007 [2] but has already been found in several countries, including the United States of America and China [3, 10, 11]. Previous studies in *P. aeruginosa* revealed that the *bla*_*KPC-2*_ gene is mobilized into only one copy of Tn*4401* (complete or truncated forms) within the two plasmid backbones. However, comparison of the genetic structures surrounding Tn*4401b* in the 24Pae112 isolate indicate to us that its incorporation was not produced by the chromosome integration of any two previously identified Inc-P or Inc-U plasmids in Colombia (Fig. 6) [15]. Conversely, the Tn*4401b* acquisition occurred by transposition at two different, but nearby sites. As far as we know, this is the first report of a double chromosomal *bla*_KPC-2_–harbouring Tn*4401* insertion in *P. aeruginosa.* Even though this duplication is a rare genetic event, it was previously reported within a 70.6-kb plasmid from a *Klebsiella pneumoniae* S9 strain identified in New York [63]. Although the Tn*4401* duplication could provide higher production of the KPC enzyme, it is necessary to determine whether it provides any advantage in the carbapenem hydrolysis and consequently an increase in the carbapenem MICs.

The origin of Tn*4401b* in the 24Pae112 isolate is unknown, although it may be related to the process of intercellular mobility (the MGE may be excised from one location and to undergo replicative transposition before to be reintegrated elsewhere in the genome) [64] from a plasmid. The 24Pae112 isolate probably acquired a *bla*_*KPC-2*_–harbouring plasmid from a *Klebsiella pneumoniae* isolate that favoured Tn*4401*b transposition to the chromosome, given that the KPC-producing *Klebsiella pneumoniae* clone is already endemic in Colombia. This Tn*4401b* mobilization from the plasmid to the chromosome has already been reported in *Klebsiella pneumoniae* [63].

Our comparative genomic analysis shows a relatively high variability in the accessory genome of ST235 isolates, suggesting great genome plasticity in this clone. This genomic flexibility could contribute to the fact that the ST235 clone can survive in the most hostile environments. The variability in the accessory genome is produced for the differential acquisition of diverse MGE, such as prophages, genomic islands and transposons (in some cases moving resistance genes), and for changes within them produced by genetic recombination events, duplications and deletions mediated by transposases and recombinases [64]. In the case of the 24Pae112 isolate, this was probably necessary for several genetic events to occur before the double Tn*4401* insertion was done. First, the acquisition of PAGI-17 by different genetics lineages, including an ST235 common ancestor because it is in almost all the ST235 isolates. Second, the insertion of a 24-kb fragment within the 5′ region of PAGI-17, this is not a genetic phenomenon exclusively associated with the ST235 clone, given that it was only found in 24Pae112. However, it is also a rare event in *P. aeruginosa* because it was only found in five isolates belonging to different genetic lineages (ST386, ST299, ST260, ST2619, and ST313). Third, the acquisition and maintenance of a MGE (plasmid) harbouring a Tn*4401*b transposon, and fourth, the transposition of the Tn*4401* from MGE to the chromosome within a site that did not affect the essential genes for the bacteria, such as PAGI-17.

From the clinical point of view, the great genomic plasticity of *P. aeruginosa* and its extraordinary ability to incorporate several copies of resistance genes generates an elevated production of B-lactam antibiotic-degrading enzymes; this limits the carbapenem use and pose the urgent need to find different treatment strategies, such as the use of combined antibiotics and the development of new therapeutic technologies. Accordingly, knowing in detail the mechanisms of bacterial resistance is an important factor to contain and prevent this great problem. For other hand, the 24Pae112 isolate was recovered from a remitted patient, who had a healthcare-associated bloodstream infection, suggesting that this *P. aeruginosa* clone could be already circulating in other regions of the country. Understanding the dynamics of the transfer of elements that confer resistance is one of the important factors to design the best practices to prevent the spread of multidrug resistant bacteria and healthcare-associated infections.

## Conclusion

Our findings together with those previously reported in the ST235 clone show the extraordinary genomic plasticity of *Pseudomonas aeruginosa*, quality that allows it to acquire, keep and modify foreign DNA into its genome. This DNA acquisition contributes to more genetic armament to face new hostile environmental conditions, such as high concentrations of antibiotics. Our findings also indicate the essential role that genomic islands play in the constant and successful evolution of *P. aeruginosa.* This has been demonstrated here with the PAGI-17, but it has also been demonstrated for PAGI-1 and -2 [22] because they serve as both catchers and natural “warehouses” of foreign DNA storage, where this bacterium can keep virulence and resistance genes with few secondary effects on its genome stability.

## Additional files

Additional file 1: **Table S1.** Characteristics of the *P. aeruginosa* ST235 genomes included in the phylogenetic tree and the genomic comparative analysis. (XLSX 16 kb)

Additional file 2: **Figure S1.** Genetic relationship by PFGE of the carbapenem-resistant *P. aeruginosa* isolates identified in the study. The black line corresponds to a genetic relationship of 80% of similarity. Numbers in the colour boxes indicate to the main PFGE pulsotype found. ST: Sequence type. The abbreviations in the susceptibility profile: TZP: piperacillin/tazobactam, CAZ: ceftazidime, MEM: meropenem, DOR: doripenem, IMP: imipenem, STX: trimethoprim/sulfamethoxazole, CIP: ciprofloxacin, AK: amikacin, GEN: gentamicin y COL: colistin. The isolate marked with asterisk (*) was selected to whole genome sequencing. (TIF 445 kb)

## Abbreviations

CLSI: Clinical and Laboratory Standards Institute
GI: Genomic islands
ICU: Intensive care unit
IS: Insertion sequence
KPC: *Klebsiella pneumoniae* carbapenemase
LGT: Lateral gene transfer
MDR: Multidrug-resistant
MGE: Mobile genetics elements
MIC: Minimum inhibitory concentration
MLST: Multilocus sequence typing
ORF: Open reading frame
PAGI: *Pseudomonas aeruginosa* genomic islands
PCR: Polymerase chain reaction
PFGE: Pulsed-field gel electrophoresis
SNP: Single nucleotide polymorphism
ST: Sequence type
VIM: Verona Integron-encoded Metallo-β-lactamase
WGS: Whole-genome sequencing
