# Supplementary figures and images for: Genome plasticity favours double chromosomal Tn4401b-blaKPC-2 transposon insertion in the Pseudomonas aeruginosa ST235 clone
Source: BMC Microbiol. 2019 Feb 20;19:45. doi: 10.1186/s12866-019-1418-6 (PMC6381643; doi:10.1186/s12866-019-1418-6)

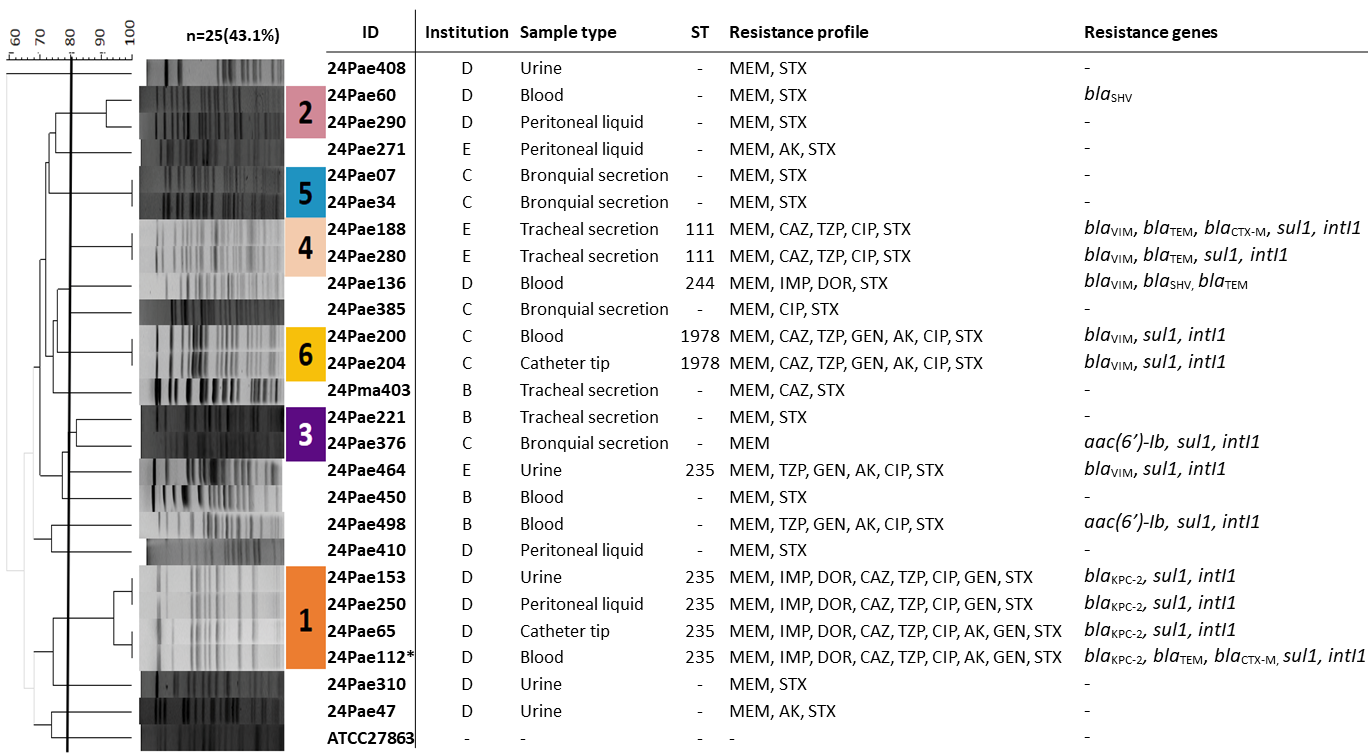

Supplement: Supplementary file 2 — Figure S1. Genetic relationship by PFGE of the carbapenem-resistant P. aeruginosa isolates identified in the study. The black line corresponds to a genetic relationship of 80% of similarity. Numbers in the colour boxes indicate to the main PFGE pulsotype found. ST: Sequence type. The abbreviations in the susceptibility profile: TZP: piperacillin/tazobactam, CAZ: ceftazidime, MEM: meropenem, DOR: doripenem, IMP: imipenem, STX: trimethoprim/sulfamethoxazole, CIP: ciprofloxacin, AK: amikacin, GEN: gentamicin y COL: colistin. The isolate marked with asterisk (*) was selected to whole genome sequencing. (TIF 445 kb) [file 12866_2019_1418_MOESM2_ESM.tif]
